# Revisiting Antagonist Effects in Hypoglossal Nucleus: Brainstem Circuit for the State-Dependent Control of Hypoglossal Motoneurons: A Hypothesis

**DOI:** 10.3389/fneur.2015.00254

**Published:** 2015-12-01

**Authors:** Victor B. Fenik

**Affiliations:** ^1^Department of Veterans Affairs Greater Los Angeles Healthcare System, Los Angeles, CA, USA; ^2^Websciences International, Los Angeles, CA, USA

**Keywords:** OSA, norepinephrine, serotonin, GABA, glycine, carbachol, REM sleep, motor control

## Abstract

We reassessed and provided new insights into the findings that were obtained in our previous experiments that employed the injections of combined adrenergic, serotonergic, GABAergic, and glycinergic antagonists into the hypoglossal nucleus in order to pharmacologically abolish the depression of hypoglossal nerve activity that occurred during carbachol-induced rapid-eye-movement (REM) sleep-like state in anesthetized rats. We concluded that noradrenergic disfacilitation is the major mechanism that is responsible for approximately 90% of the depression of hypoglossal motoneurons, whereas the remaining 10% can be explained by serotonergic mechanisms that have net inhibitory effect on hypoglossal nerve activity during REM sleep-like state. We hypothesized that both noradrenergic and serotonergic state-dependent mechanisms indirectly control hypoglossal motoneuron excitability during REM sleep; their activities are integrated and mediated to hypoglossal motoneurons by reticular formation neurons. In addition, we proposed a brainstem neural circuit that can explain the new findings.

## Introduction

The atonia of skeletal muscles is an insignia that distinguishes rapid-eye-movement (REM) sleep from the other two states of mammal existence, such as non-REM (NREM) sleep and wakefulness. It is an important evolutional adaptation that prevents potentially harmful unconsciousness and uncoordinated motor activity during REM sleep that includes movements of the body and its extremities, as well as chewing, tongue movements, etc. The importance of the REM sleep muscle atonia becomes obvious when inadequate muscle paralysis during REM sleep results in the “dream enactment behavior” in patients with REM sleep behavioral disorder (RBD) (1–3). The RBD is currently treated with benzodiazepines from which clonazepam is the most effective to reduce the generation of REM sleep phasic events by enhancing pontine GABA inhibition not affecting the REM sleep atonia (1, 4, 5).

Pharyngeal muscles also become relaxed during NREM and REM sleep and the sleep-related reduction of their tonus may lead to a partial or complete closure of airway in obstructive sleep apnea (OSA) patients, which have relatively narrow pharyngeal orifice due to anatomical abnormalities or obesity (6, 7). Another factor that contributes to OSA pathology is the insufficient strength of the genioglossus muscles activation in response to negative pressure, which in addition is inhibited during sleep (8, 9). Activity of the genioglossus and tensor palatine is elevated in OSA patients compared to healthy controls, which helps to overcome the anatomical deficiencies and maintain airway opened during wakefulness (10). However, these neurological compensatory mechanisms are eliminated during sleep (10).

Obstructive sleep apnea patients chronically experience repetitive nocturnal periods of hypoxia and hypercapnia (11–13). The obstruction episodes usually end by awakenings that restore airway patency at the expense of the sleep continuity and the total sleep (14–16). OSA is a growing sleep disorder (17, 18). OSA patients suffer from excessive daytime somnolence, impaired learning and psychomotor vigilance, and overall decrease in quality of life (16, 19–24). The OSA is also linked to hypertension (11–13, 24, 25) and an increase of risk for stroke and death (26). The treatment options for OSA are mostly limited to the weight loss and use of oral appliances that include the positive air pressure therapy, surgical procedures, and the electrical stimulation of hypoglossal nerve and upper airway muscles, whereas no effective pharmacological treatment is available (16, 27–31).

Hypoglossal motoneurons that innervate pharyngeal and tongue muscles (32) play a key role in the maintenance of upper airway muscle tone (30). The importance of the activity of hypoglossal motoneurons for the maintenance of pharyngeal airway patency is further supported by the findings that an electrical stimulation of hypoglossal nerve during sleep eliminates hypopnea/apnea events in selected groups of OSA patients (29, 31). Therefore, the emerging understanding of the neurochemical mechanisms that are responsible for the depression of hypoglossal motoneuron activity during NREM and REM sleep may provide valuable information for developing pharmacological treatments for OSA.

We would like to review and provide new insights into findings that were obtained in our previous experiments that employed the injections of the mixtures containing adrenergic, serotonergic, GABAergic, and glycinergic antagonists into the hypoglossal nucleus in order to pharmacologically abolish the depression of hypoglossal nerve activity during carbachol-induced REM sleep-like episodes in anesthetized rats (33–35). In addition, we propose a hypothetical brainstem neural circuit that can explain the original data and their new interpretations.

## Methodological Considerations

**Figure 1:**
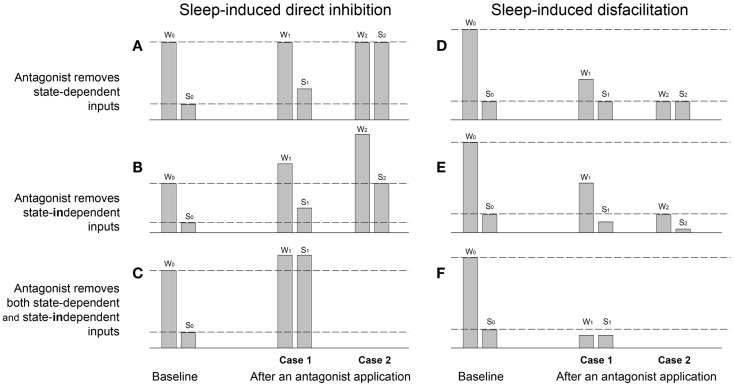
**Theoretical examples of sleep-induced inhibitory (A–C) and disfacilitatory (D–F) effects on the magnitude of a parameter value measured during wakefulness (W) and sleep (S) before (baseline W0,S0) and after applications of antagonists (Cases 1 and 2), which supposedly remove either state-dependent inhibitory (A) and facilitatory (D) inputs, or tonic state-independent inhibitory (B) and facilitatory (E) inputs or simultaneously state-dependent and state-independent inhibitory (C) and facilitatory (F) inputs**. W0 and S0 – the parameter value measured during baseline wakefulness and sleep, respectively; W1 and S1 – the Case 1 after an antagonist application during wakefulness and sleep, respectively; W2 and S2 – the Case 2 after an antagonist.

Since effects of the applied antagonists on the activity of motoneurons during wakefulness, NREM, and REM sleep are heterogeneous and sometimes difficult to interpret, we would like to theoretically summarize and provide interpretations of the most common outcomes of antagonist applications on a parameter value that is measured during, e.g., sleep and wakefulness (Figure 1). The parameter is either the amplitude of membrane potential, or frequency of neuronal firing rate, or amplitude of moving average of nerve activity, etc. In these theoretical examples, sleep has a depressant influence on the magnitude of the measured parameter and, therefore, its value is decreased during sleep as compared to wakefulness due to either direct inhibition of motoneurons (Figures 1A–C) or the removal of excitatory inputs from motoneurons, i.e., disfacilitation (Figures 1D–F). In addition, applied antagonists may block receptors that are relevant to the effect of sleep and, thereby, reduce the sleep effects (Figures 1A,D), or they may block some other receptors that do not mediate the sleep effects but that mediate tonic excitatory or inhibitory drives that affect motoneuronal excitability (Figures 1B,E).

The Figure 1A demonstrates examples of the parameter value measured during baseline wakefulness (W) and sleep (S) before an antagonist application (W0,S0). The Case 1 (W1,S1) shows an example of a partial removal of sleep-induced direct inhibition of motoneurons by the antagonist application, i.e., “state-dependent disinhibition.” Note that the characteristic for the state-dependent disinhibition is the reduction of the ratio S1/W1 after antagonists as compared to the baseline ratio S0/W0 by increasing the parameter value during sleep (S1), whereas its value during wakefulness (W1) remains similar to the baseline value (W0). The partial antagonist effects (W1,S1) show inability of an antagonist to fully remove the inhibition, which could be explained by different factors such as contributions of additional inhibitory neurotransmitters/receptors that are not affected by the applied antagonist, or incomplete covering of distant receptors due to insufficient antagonist volume/dose/concentration, or incomplete antagonism due to insusceptibility of relevant receptors for the antagonist, etc. By contrast, the full disinhibition (Case 2: W2,S2) is conclusive, and it can be only interpreted as a complete removal by an antagonist of the inhibitory input that is solely and fully responsible for the decrease in the parameter value during sleep as compared to wakefulness.

The Figure 1B shows two examples (Cases 1 and 2) of the removal by an antagonist of a tonic not state-dependent inhibition. The main outcome of such a “state-*in*dependent disinhibition” is the increase in values of a measured parameter during both wakefulness (W1) and sleep (S1) as compared to its values in the baseline (W0,S0). In addition, since no state-dependent input is removed, the ratio S1/W1 is approximately equal to the baseline ratio S0/W0. The Case W2,S2 shows an example of the removal of a much stronger tonic inhibitory input so that the parameter value during sleep S2 become equal to its value during wakefulness in control W0. The ratios of the parameter values after the antagonist (S1/W1 or S2/W2) could be used to suggest removal of additional sleep-dependent inhibitory inputs if they significantly deviate from the baseline ratio S0/W0.

The Figure 1C shows an example of simultaneous removal by an antagonist of state-dependent and state-*in*dependent inhibitions. The similarity of the parameter values after antagonist during both sleep (S1) and wakefulness (W1) indicates the full state-dependent disinhibition, whereas the increasing in values of S1 and W1 relative to the W0 value suggests the additional removal of state-*in*dependent inhibitory inputs.

The Figure 1D demonstrates two cases of the antagonist-induced reduction of the “state-dependent disfacilitation,” which occurs when the antagonist removes the same excitatory inputs that are removed during sleep. In case of the partial antagonist effect, the decrease in ratio S1/W1 relative to the baseline ratio S0/W0 is due to the decrease of the parameter value during wakefulness (W1), whereas its value during sleep after antagonist (S1) remains similar to its baseline sleep value (S0). The Case W2,S2 shows a complete state-dependent disfacilitation. In this case, all state-dependent excitation, which is normally removed by sleep (S0), is removed by the antagonist and, therefore, the parameter value W2 is equal to its value during S0. Unlike the partial antagonism, the complete disfacilitation conclusively suggests that receptors, which were blocked by the antagonist application, are fully responsible for the disfacilitatory state-dependent effect of sleep.

The Figure 1E provides two examples of the removal of a tonic not state-dependent excitation by an application of an antagonist. The characteristic feature of this antagonist-induced “state-*in*dependent disfacilitation” is that both parameter values after antagonist during both wakefulness (W1) and sleep (S1) decreased as compared to their magnitudes during baseline conditions W0 and S0, respectively. The Case W2,S2 shows an example of the removal of a stronger sleep-*in*dependent excitation so that the parameter value W2 is decreased to S0. Also similar to the state-*in*dependent disinhibition (see Figure 1B), the state-*in*dependent disfacilitation is characterized by both ratios S1/W1 and S2/W2 being approximately equal to the baseline ratio S0/W0. Otherwise, a significant difference between these ratios and the baseline ratio would indicate an additional removal of state-dependent inputs.

The Figure 1F shows an example of a complete removal of a state-dependent excitation by an antagonist application, which is evidenced by S1 = W1, and a simultaneous removal of a state-*in*dependent excitation, which reduces both values W1 and S1 below the baseline value S0.

The discussed examples of antagonist modulations of the sleep effects on a measured parameter provide simplified but useful interpretations, which may help to uncover underlying mechanisms of the antagonist effects in real antagonist experiments, e.g., state-dependent or state-*in*dependent removal of inhibitory or excitatory inputs. The important outcome is that during the antagonist-induced state-dependent disinhibition, the S1/W1 ratio is decreased due to the increase in S1 value, whereas during state-dependent disfacilitation, the S1/W1 ratio is decreased due to the decrease in W1 value. Also, only cases with the complete disinhibition or disfacilitation can be regarded as fully conclusive, whereas the cases with partial effects could have different interpretations. Naturally, to achieve the complete effects of applied antagonists, they have to cover and block all receptors that mediate a studied function.

## REM Sleep Animal Models to Study Neurotransmitter Mechanisms of REM Sleep-Related Depression in Different Pools of Motoneurons

Carbachol animal models of REM sleep have been successfully used for decades to study mechanisms of both REM sleep generation and REM sleep-related muscle atonia, in conjunction with the naturally sleeping behaving animals [reviewed by Kubin (36)]. Carbachol, a dual muscarinic and nicotinic cholinergic agonist, injected into dorsolateral pontine tegmenum in cats or into the homologous region in rats (the sub-Locus Coeruleus region or the sub-laterodorsal nucleus) mimics the increased cholinergic drive in these regions during natural REM sleep and, therefore, elicits REM sleep-like state (REMSLS) in decerebrated or anesthetized animals. The REMSLS have many features of natural REM sleep, including atonia of postural and orofacial muscles, which make these model useful to study neurochemical mechanisms of the muscle atonia. The REMSLS may also be triggered by pontine injection of bicuculline, a GABA_A_ receptor antagonist (37, 38), or by pontine electrical stimulation (39). When anesthetized animals are used to produce REMSLS, there is always a concern that anesthetic may affect the experimental outcomes. However, the outstanding stability and repeatability of anesthetized preparations, which allows performing complex *in vivo* experiments with precise temporal dissection of investigated phenomena under highly controlled conditions, make their use often more advantageous over behaving animals. Obviously, important findings and conclusions obtained in anesthetized animals have to be confirmed in behaving animals.

## The Neurotransmitter Mechanisms of REM Sleep-Related Depression of Spinal, Trigeminal, and Hypoglossal Motoneurons

### Spinal Motoneurons

The neurotransmitters that are involved in the REM sleep-induced muscle atonia were first conclusively demonstrated in the pioneering work conducted in Michael Chase’s laboratory (40). By employing the intracellular recording from lumbar motoneurons in behaving cats, authors determined that the REM sleep-induced atonia of skeletal muscles is due to postsynaptic inhibition of spinal motoneurons mediated by glycine. Indeed, iontophoretically applied strychnine, a glycinergic antagonist, abolished all important REM sleep-induced features that contribute to the motoneuron deactivation during REM sleep: (1) hyperpolarization of the motoneuron membrane that increases the threshold for the action potential generation; (2) decrease in input resistance that shunted summation of sub-threshold excitatory postsynaptic potentials; and (3) the increase in rheobase that is a principal measure of neuronal excitability. Since both membrane hyperpolarization and increase in membrane conductance contribute to increase in rheobase, the abolition of the REM sleep-related increase in rheobase by strychnine is the major finding obtained in this study indicating that the reduction of excitability of lumbar motoneurons is due to postsynaptic inhibition mediated by glycine. In addition, relatively large spontaneous sub-threshold inhibitory postsynaptic potentials (IPSPs) were observed during NREM and with fourfold higher frequency during REM, sleep (40). The amplitude and frequency of these IPSPs were significantly reduced by strychnine during both NREM and REM sleep suggesting their glycinergic nature (40, 41). However, the causal relationship was not demonstrated between the appearance of the IPSPs and the decrease in motoneuron excitability in these studies. In addition during carbachol-induced REMSLS, the appearance of the IPSPs did not cause a significant membrane hyperpolarization in either spinal (42) or hypoglossal motoneurons (43). Furthermore, it has been reported that the IPSPs are closely associated with ponto-geniculo-occipital (PGO) waves (44, 45), which are regarded as the “phasic” REM sleep events that have different generation mechanisms compared to the “tonic” REM sleep events, such as muscle atonia (46). For example, the occurrence of PGO waves did not correlate with the loss of muscle atonia produced by the lesions of the dorsal pontine tegmentum in cats (47).

### Trigeminal Motoneurons

Similar IPSPs were found during intracellular recordings in trigeminal motoneurons during REM sleep (48). These IPSPs were also strychnine sensitive and their appearance strongly correlated with PGO waves (49, 50). However, in recent studies, antagonizing of glycine and GABA_A_ receptors in the motor trigeminal nucleus did not abolish REM sleep-related depression of masseter muscle EMG in behaving rats (51, 52). Likewise after the combined antagonism of GABA_A_, GABA_B_, and glycine receptor in the motor trigeminal nucleus, the relative REM sleep-induced depression of trigeminal motoneurons remained similar to that before antagonists, even though the antagonists increased the masseter muscle activity during REM sleep to the level that was observed during NREM sleep before the antagonists (53). On the other hand, there is convincing evidence that withdrawal of glutamate excitation is responsible for depression of trigeminal motoneurons during NREM sleep relative to wakefulness, but not during REM sleep (54). Also, the interaction between noradrenergic and glutamatergic signaling has been recently described at the trigeminal motor nucleus (55). Thus, neurotransmitter mechanisms of REM sleep-induced depression of trigeminal motoneurons remain uncertain.

### REM Sleep-Induced Hypoglossal Motoneuron Depression

The mechanisms of REM sleep-induced hypoglossal motoneuron depression (REM-HD) were studied using intracellular recording during carbachol-induced REMSLS in anesthetized and decerebrated cats (43). The main finding of this study was that the rheobase of hypoglossal motoneurons significantly increased by 60% during REMSLS. Also, similar to spinal and trigeminal motoneurons, large strychnine-sensitive IPSPs appeared in hypoglossal motoneurons during REMSLS. Based on this similarity and indications that they are strychnine sensitive, authors concluded that postsynaptic inhibition was responsible for the decrease in hypoglossal motoneuron excitability during REMSLS. However, statistical analyses were not provided for the strychnine sensitivity of these IPSPs. Also surprisingly, effects of strychnine on the magnitude of rheobase were not described, which would provide direct evidence that glycinergic inhibition causes the reduction of motoneuron excitability (43).

Additional analyses of changes in motoneuron electrophysiological properties (56) and in field potentials evoked by stimulation of ipsilateral hypoglossal nerve (57) were reported in subsequent studies of hypoglossal motoneurons during carbachol-induced REMSLS. However, strychnine was not used in those studies to confirm the glycinergic nature of the observed phenomena. Also, the behavior of the IPSPs and rheobase in hypoglossal motoneurons was described during natural sleep and wakefulness, although no antagonist was used in that study to prove the inhibitory effects (58). Thus, the hypothesis regarding the key role of glycinergic inhibition in REM-HD is only based on observations that glycinergic IPSPs appear during both natural REM sleep and carbachol-induced REMSLS. The assumption that they lead to the decrease of excitability of hypoglossal motoneurons during REM-HD was not experimentally tested. Also, no attempts were made to verify if the decrease of excitability of hypoglossal motoneurons during REM-HD was strychnine sensitive (e.g., increase in rheobase, membrane hyperpolarization, or decrease in membrane resistance). In addition, “extracellular” studies, in which the activity in hypoglossal nerve or genioglossal muscle was measured, did not provide evidence regarding a role of glycine in REM-HD during carbachol-induced REMSLS in either decerebrated cats (59) or anesthetized rats (33, 34). Furthermore, no contribution of either GABA or glycine was found to REM-HD in behaving rats (60).

The more conclusive results regarding neurotransmitter control of excitability of hypoglossal motoneurons during REM-HD were obtained using carbachol model of REM sleep in anesthetized rats. The antagonism of noradrenergic and serotonergic receptors within and around hypoglossal nucleus abolished REM-HD that occurred during carbachol-induced REMSLS in anesthetized rats (35). These findings provide a strong support for the hypothesis that aminergic disfacilitation plays a key role in REM-HD. Quantitatively, contribution of noradrenaline to REM-HD was much stronger compared to 5HT. Importantly, the significant involvement of noradrenaline in REM-HD was experimentally demonstrated in behaving rats (61). Since the serotonin contribution to REM-HD was relatively small (35), it was not observed in behaving rats (62) probably due to the inheritably higher variability of results obtained in behaving animals.

Other neurotransmitters, such as glutamate (63, 64), presynaptically acting acetylcholine (63), and GABA (65), have been implicated in the mechanisms of REM-HD. Recently, a relatively strong effect of the antagonism of muscarinic receptors within hypoglossal nucleus on the genoiglossus muscle activity was found during wakefulness, NREM sleep, and REM sleep in behaving rats (66). The effectiveness of this antagonism was more pronounced during REM sleep, which suggested that postsynaptic cholinergic inhibition may also contribute to REM-HD (66).

### Conclusion

Thus, to date, the abolition of REM sleep-related depression of motoneurons, which is achieved by application of antagonists and can be regarded as the “gold standard” to define the responsible neurotransmitters, has been demonstrated for two pools of motoneurons – spinal and hypoglossal. First of all, this indicates that different neurotransmitters mediate REM-induced depression of muscle tone in different motoneurons. Second, those studies conclusively showed that (1) REM sleep-induced depression of *spinal* motoneurons is due to postsynaptic inhibition mediated by glycine (40) and (2) REM sleep-induced depression of *hypoglossal* motoneurons is due to withdrawal of noradrenergic and serotonergic drives (35). The latter has been determined during REMSLS in anesthetized animals (35) and confirmed in behaving animals (61).

## Revisiting Antagonist Effects on Hypoglossal Nerve Activity During Carbachol-Induced Remsls in Anesthetized Rats

We have conducted a series of experiments with a goal to determine the neurochemical mechanisms of the depression of hypoglossal motoneurons during REMSLS using a carbachol model of REM sleep in anesthetized rats (33–35). The REMSLS were triggered by injections of carbachol into pontine dorsal sub-coeruleus region (or sub-laterodorsal nucleus) (38). The carbachol injections repeatedly elicited many features of natural REM sleep that included activation of EEG (appearance of the high frequency in 6–12 Hz band without a decrease in EEG amplitude), hippocampal theta rhythm, depression of hypoglossal nerve activity, and silencing of LC and A5 neurons (36, 67, 68). Only some features of natural REM sleep (the desynchronization of EEG and the REM sleep phasic events with the increased respiratory rate) were not observed during REMSLS in this model due to anesthesia. However, the high stability of this preparation and excellent repeatability of the magnitude of the decrease in inspiratory hypoglossal nerve activity during REMSLS, which is analogous to REM-HD, allowed us to test effects of various antagonists on the REM-HD. Antagonists were injected into ipsilateral hypoglossal nucleus by three 40 nL injections that were evenly placed along the hypoglossal nucleus to ensure that all hypoglossal motoneurons are covered with antagonists right after the end of the injections.

**Figure 2:**
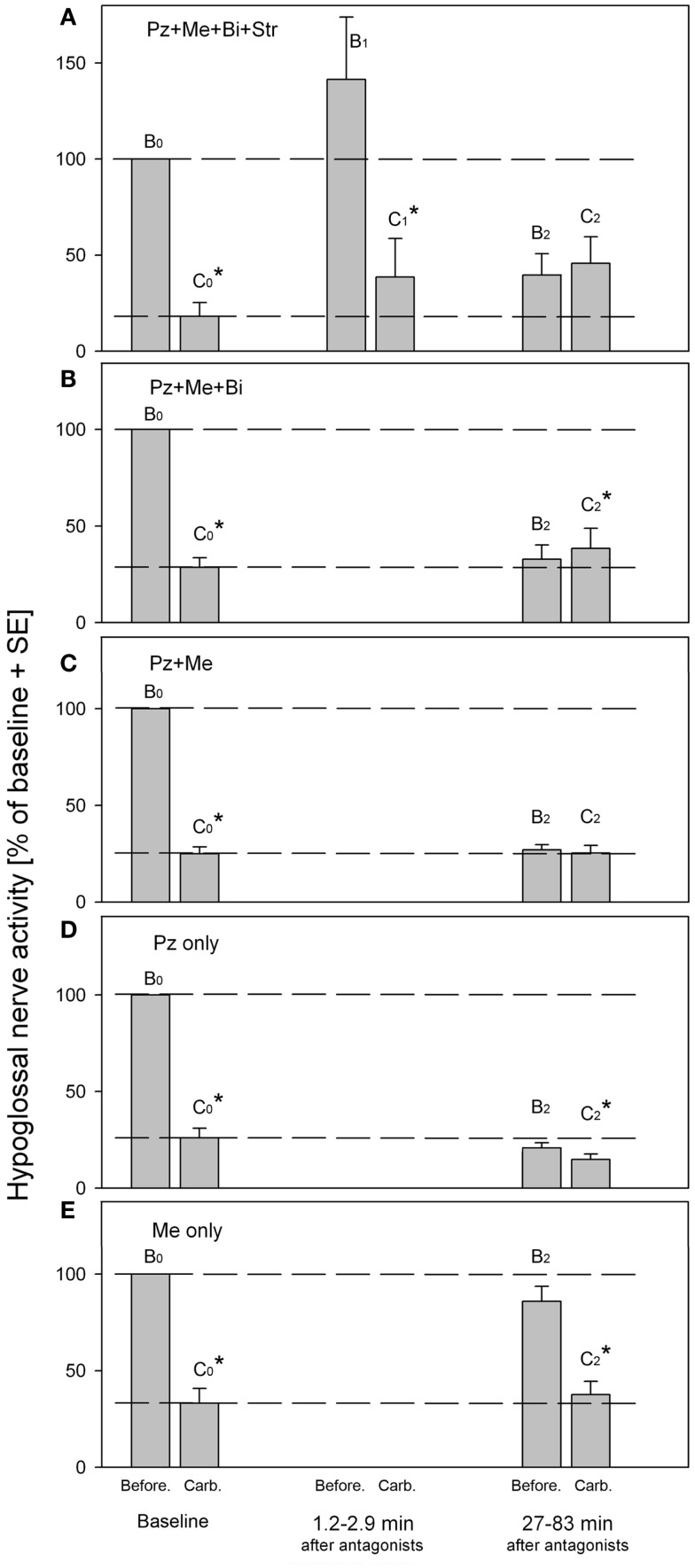
**Summary of hypoglossal nerve activity during carbachol- induced REM sleep-like episodes that were evoked before (baseline B0,C0) and at different times after (B1,C1 and B2,C2), the injections of the antagonist combinations into hypoglossal nucleus**. **(A)** Combined injections of prazosin, methysergide, bicuculline and strychnine. **(B)** Combined injections of prazosin, methysergide and bicuculline. **(C)** Combined injections of prazosin and methysergide. **(D)** Injections of prazosin only. **(E)** Injections of methysergide only. Pz, prazosin, an α1-adrenoceptor antagonist; Me, methysergide, a broad-spectrum serotonergic antagonist; Bi, bicuculline, a GABA_A_ antagonist; Str, strychnine, a glycinergic antagonist. B0 and C0, baseline hypoglossal nerve activity measured before and during carbachol, respectively; B1 and C1, hypoglossal nerve activity during “early” carbachol responses; B2 and C2, hypoglossal nerve activity during “late” carbachol responses. *p < 0.05, paired *t*-test [adapted from Fenik et al. (33–35)].

The Figure 2 summarizes the effects of antagonists on the REM-HD that were obtained in our series of experiments (33–35). The amplitudes of moving average of hypoglossal nerve activity were measured before (B) and during carbachol (C) at baseline (B0,C0), right after (B1,C1) and at approximately 1 h after (B2,C2), the antagonist injections into hypoglossal nucleus. Additional episodes of REMSLS were elicited by carbachol at later times to observe the recovery process (not shown).

The Figure 2A shows the results of injections of the antagonist mixture that contained the solution of the following four antagonists in saline: 1 mM of strychnine, a glycinergic receptor antagonist; 1 mM of bicuculline, a GABA_A_ receptor antagonist; 0.2 mM of prazosine, an α1-adrenoceptor antagonist; and 1 mM of methysergide, a broad-spectrum serotonergic (5HT) receptor antagonist (33). The effectiveness of each antagonist in the mixture was verified in separated experiments. The injections of the antagonist mix into hypoglossal nucleus resulted in immediate increase of hypoglossal nerve activity due to blockade of glycine and GABA_A_ receptors (see B1 in Figure 2A). We expected that after the blockade of the four types of receptors, which were implicated in the mediation of REM-HD and targeted by the antagonist mix, the REM-HD would be abolished during the early REMSLS (B1,C1). However, the REM-HD was still very prominent and the ratio of the amplitudes of the nerve activity before and during carbachol in the early REMSLS was C1/B1 = 27%, similar to baseline C0/B0 = 18%. The failure of the antagonist mix to abolish the REM-HD early after the antagonists cannot be explained by partial covering of the relevant receptors because we specifically calculated the number, placement, and volume of the injections to be able to cover all motoneurons by the antagonists immediately after the end of the antagonist injections. In addition, according to the analysis of the diffusion of the antagonists from their injection sites, the effective diameter of the antagonist injections after 10 min following their injection (the injection time 8.2 min plus the time when the measurements B1 and C1 were taken 1.2–2.9 min after the end of the injections) would be approximately twice larger than the diameter of the hypoglossal nucleus (34). Furthermore, our previous experience with injections of similar concentrations of glycine, GABA_A_, α1-adrenoceptor or 5HT receptor agonists, and antagonists into the hypoglossal nucleus in the same preparations, suggested that antagonists blocked the effects of either endogenous or exogenous ligands within 1 min after injections. Therefore, the failure of the antagonist mix to abolish the REM-HD early after the antagonists cannot be explained by insufficient timing that may be required to block the relevant receptors within the hypoglossal nucleus.

Thus, the receptors that were blocked by the mixture of the four antagonists within the hypoglossal nucleus are not responsible for the REM-HD. Similar conclusions regarding the involvement of glycinergic, GABAergic, and 5HT receptors in REM-HD were made in behaving rats (60, 62). In addition according to our previous experience, the removing of endogenous adrenergic excitation by prazosin injections into hypoglossal nucleus did not reduce the spontaneous activity in hypoglossal nerve activity in anesthetized rats (69). Likewise, blocking 5HT receptors by iontophoretically applied methysergide did not change the spontaneous firing rate of extracellularly recorded hypoglossal motoneurons in decerebrate cats (70). Thus, the spontaneous excitatory noradrenergic and 5HT drives within the hypoglossal nuclei are negligible and, therefore, their withdrawal during REM sleep-like effects cannot explain the REM-HD in these preparations.

However, when carbachol was injected at 27–83 min after the end of the antagonist injections (Figure 2A, B2,C2), the REM-HD was abolished. Interestingly, an additional excitatory component appeared in hypoglossal nerve activity after carbachol injection C2. We explained the abolition of the REM-HD during the late carbachol injections B2,C2 by the diffusion of the antagonists outside the hypoglossal nucleus and blocking the relevant receptors within the reticular formation (34).

The behavior of the antagonist mix without strychnine (Figure 2B) was very similar to the full mixture, suggesting that the antagonism of glycine receptors did not play a role in the abolition of REM-HD during the late carbachol injections. The small excitation was also present during late carbachol injections (Figure 2B, C2). When these trials and the trials with the full antagonist mixture are combined, the increase in hypoglossal nerve activity during carbachol C2 (Figures 2A,B) became statistically different from the nerve activity before carbachol B2 (*p* < 0.05; *n* = 12; paired Student’s *t*-test).

When only two antagonists, such as prazosin and methysergide, were left in the mixture, the REM-HD was still abolished at the late carbachol injections (Figure 2C, B2,C2). Since the trials with prazosin or methysergide alone failed to abolish the REM-HD (Figures 2D,E), the antagonism of α1-adrenoceptors and 5HT receptors is necessary and sufficient to abolish the REM-HD in this preparation. In these trials during late carbachol injections (B2,C2), there was no excitatory component, which suggests that the blocking of GABA_A_ receptors was responsible for its appearance when bicuculline was present in the antagonist mix.

The abolition of the REM-HD by the mix containing only prazosin and methysergide is a typical example of the complete disfacilitation (see Figure 1D, W2,S2). One conclusion could be made that prazosin removed some adrenergic REM sleep-dependent excitatory input to hypoglossal motoneurons, whereas methysergide removed the remaining REM sleep-dependent excitation mediated by serotonin. Therefore, in order to quantify the contribution of each adrenergic and 5HT contribution to the state-dependent excitation of hypoglossal motoneurons, we conducted two separated trials with injections of prazosin alone and methysergide alone into hypoglossal nucleus.

Unexpectedly, the “prazosin only” injections reduced the level of spontaneous activity in hypoglossal nerve B2 at the time of the late carbachol-induced REMSLS (Figure 2D) approximately to the level of the nerve activity during baseline carbachol responses C0. Thus, all excitatory inputs to hypoglossal motoneurons, the removal of which was necessary for the full disfacilitation, were removed by prazosin alone. However, a small but statistically significant [*p* < 0.05, *n* = 6 (35)] depression of hypoglossal nerve activity was still present during the late carbachol responses (B2,C2) and the level C2 decreased below the level C0 of the nerve activity. Thus, when methysergide was present in the mix with prazosin, it increased level C2 to the level B2. Such an effect of methysergide is in accordance with the removal of state-dependent inhibitory inputs to hypoglossal motoneurons, similar to the cases of disinhibition in Figure 1A.

From the trials with prazosin only injections, it is possible to quantitatively estimate the relative contributions of the noradrenergic and serotonergic mechanisms to REM-HD. Prazosin decreased spontaneous activity in hypoglossal nerve from B0 = 100% to B2 = 20.8% (Figure 2D). During the following carbachol, the nerve activity decreased to C2 = 14.8% of baseline (35). Thus, we can calculate the total magnitude of the nerve depression: 100–14.8 = 85.2(%); a noradrenergic contribution: 100–20.8 = 79.2(%); and a serotonergic contribution: 20.8–14.8 = 6(%). Thus, the estimated relative contribution of noradrenergic mechanisms to REM-HD is 79.2/85.2 × 100 = 93(%), and the estimated relative contribution of serotonergic mechanisms is 6/85.2 × 100 = 7(%).

The “methysergide only” injections had mixed effects on the activity in hypoglossal nerve (Figure 2E) that can be explained by effects of methysergide on multiple receptors (71). During the time of the late carbachol-induced REMSLS, spontaneous nerve activity B2 tended to decrease below level B0. This decrease might indicate a net state-dependent disfacilitatory effect of methysergide on REM-HD. However, the facts that prazosin alone fully disfacilitated hypoglossal nerve activity (evidenced by B2 = C0 in Figure 2D) implies that it was rather a removal of REM state-*in*dependent inputs. In addition, the level of the nerve activity during the late REMSLS (C2 in Figure 2E) tended to be higher than that during the baseline REMSLS (C0), which suggests that methysergide removed some inhibitory inputs to hypoglossal motoneurons (see Figure 1A, Case 2). This disinhibitory effect of methysergide was relatively small but the value C2 was likely to be reduced by the state-*in*dependent disfacilitation (see above and Figure 1E).

**Figure 3:**
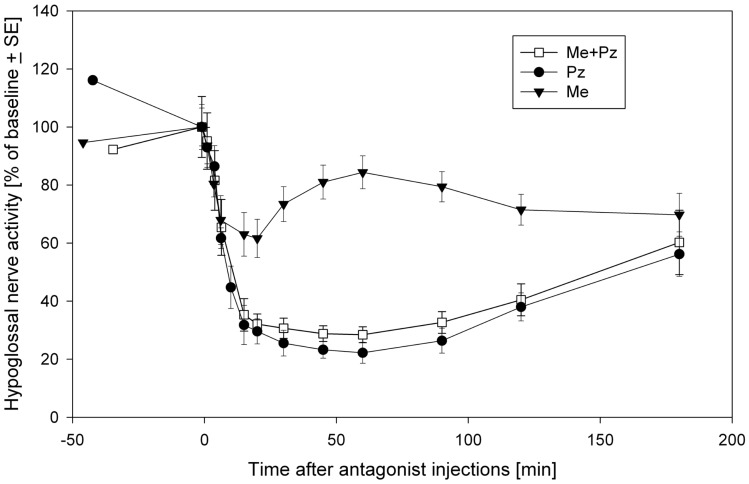
**The time-courses of spontaneous activity recorded in the hypoglossal nerve after injections of a mix containing both prazosin (Pz) and methysergide (Me) (open squares), prazosin only (filled circles) and methysergide only (filled triangles) into hypoglossal nucleus**. Modified from Fenik et al. (35) with permission of the American Thoracic Society. Copyright © 2015 American Thoracic Society.

Thus, it seems that prazosin is fully responsible for the disfacilitation, whereas methysergide for the disinhibitory effects on the REM-HD. The inhibitory role of 5HT in REM-HD is unexpected. However, this conclusion is additionally supported by the time-courses of the effects methysergide on the spontaneous activity in the hypoglossal nerve (Figure 3). During the diffusion, prazosin blocked more α1-adrenoceptors and more excitatory inputs to hypoglossal motoneurons were removed, which pushed the hypoglossal nerve activity down to its minimal level approximately at the time of the late carbachol responses (Figure 3, filled circles). The methysergide had less dramatic effects on the spontaneous nerve activity (Figure 3, filled triangles) that affected the time-course of combined antagonist effects (open squares). In addition, the time-course of methysergide effects had a bi-phasic shape. Initially, the spontaneous activity in hypoglossal nerve decreased likely due to the state-*in*dependent disfacilitation (see above), i.e., affecting receptors that likely do not contribute to REM-HD. However, at later times, hypoglossal nerve activity started to increase and reached its peak approximately at the time of the late carbachol responses (see Figure 3). This increase of hypoglossal nerve activity suggests that methysergide diffused to locations where it affected different 5HT receptors, so that its net effect was disinhibitory to hypoglossal motoneurons. We believe that these are the 5HT receptors, which mediate the depression of hypoglossal nerve activity in the “prazosin only” trials during the late carbachol-induced REMSLS (C2 in Figure 2D).

In summary, the disfacilitation of noradrenergic drive is a major mechanism of the REM-HD that is responsible for approximately 90% of REM-HD. The significant involvement of noradrenaline in REM-HD is also supported by experiments in behaving rats (61). The 5HT-related inhibition of hypoglossal motoneurons additionally contributes to approximately 10% of REM-HD. Possibly because of its relatively small magnitude and the inheritably larger data variability in behaving animals, the contribution of 5HT was not detected in behaving rats (62).

## The Neural Circuit of State-Dependent Control of Hypoglossal Motoneurons

We would like to suggest a brainstem neural circuit that can explain the state-dependent control of hypoglossal motoneurons. It contains the minimal number of neuronal pools that are necessary and sufficient to fulfill our conclusions: (1) neither glycine nor GABA_A_ receptors that are located within or near the hypoglossal nucleus are responsible for REM-HD, at least in this animal model of REM sleep; (2) there is, however, a small GABA_A_-dependent excitation of hypoglossal motoneurons during REM-HD; (3) blocking both α1-adrenergic and 5HT receptors outside the hypoglossal nucleus is necessary for the abolition of REM-HD; (4) the major mechanism of REM-HD is a noradrenergic disfacilitation of hypoglossal motoneurons; and (5) 5HT mechanisms contribute to REM-HD by net inhibitory effects on hypoglossal motoneurons.

**Figure 4:**
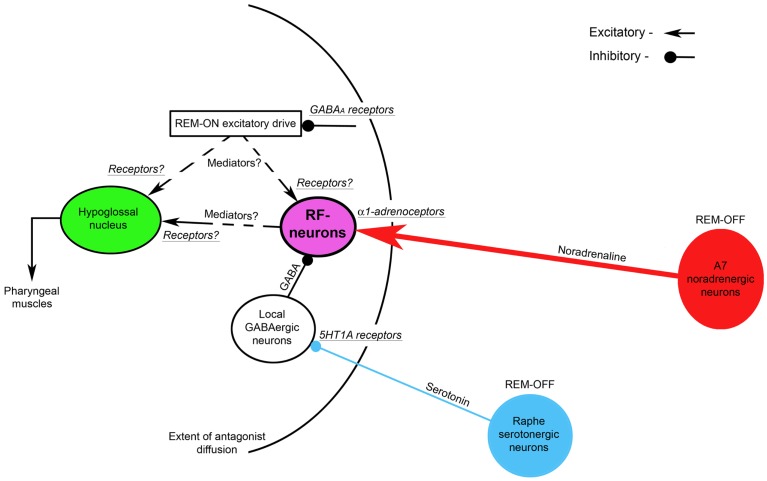
**A hypothetical brainstem circuit that illustrates the key neuronal pools, which participate in the state-dependent control of hypoglossal motoneuron excitability during REM sleep**. The reticular formation neurons (RF-neurons) integrate and mediate noradrenergic and serotonergic drives to hypoglossal motoneurons. The REM-OFF A7 noradrenergic neurons excite RF-neurons via α1-adrenoceptors. The RF-neurons are tonically inhibited by local GABAergic neurons, activity of which is controlled by REM-OFF raphe serotonergic neurons via inhibitory 5HT1 receptors. The mediators and receptors through which RF-neurons directly or indirectly excite hypoglossal motoneurons remain to be determined. A hypothetical REM-ON excitatory drives to hypoglossal and/or RF-neurons are controlled by GABA_A_ inhibitory receptors. The curved line shows the extent of the diffusion of antagonists that were injected into hypoglossal nucleus as discussed in this review.

The Figure 4 shows the suggested neural circuit. The major neuronal pools that are responsible for the state-dependent control of hypoglossal motoneurons are noradrenergic A7 neurons and hypothetical medullary reticular formation neurons (RF-neurons) that mediate the noradrenergic excitatory drive from A7 neurons to hypoglossal motoneurons (72). We propose that the A7 neurons excite the RF-neurons by activating their α1-adrenoceptors, which are located within the radius of the diffusion of the antagonists (see Figure 4) that was estimated 0.9–1.4 mm from the center of the hypoglossal nucleus (34). These RF-neurons have a net excitatory effect on hypoglossal motoneurons, but their projections to hypoglossal motoneurons (direct or indirect) and the involved neurotransmitters/receptors need to be determined in future experiments. The A7 neurons decrease their activity during REMSLS (73) and are likely to be silent during REM sleep (74). Therefore, the silencing of A7 neurons during REM sleep disfacilitates the RF-neurons and, thereby, hypoglossal motoneurons. Our hypothesis that aminergic drive affects hypoglossal motoneurons indirectly, i.e., mediated by RF-neurons, is in accordance with intracellular studies, in which no evidence for aminergic disfacilitation was found in hypoglossal motoneurons during REM sleep or REMSLS (56, 58).

To explain mechanisms of both the disinhibitory effect of methysergide on REM-HD and the resultant net inhibitory effect of 5HT neurons on hypoglossal motoneurons during REM sleep, we hypothesize that 5HT neurons, which are located in medullary and/or pontine raphe nuclei, inhibit local GABAergic neurons via 5HT1A receptors. These GABAergic neurons, in turn, inhibit the RF-neurons (see Figure 4). Since brainstem 5HT raphe neurons are REM-OFF neurons (75, 76), they inhibit the local GABAergic neurons during wakefulness and NREM sleep. Both the low activity of the GABAergic neurons and a high level of noradrenergic excitatory drive from A7 neurons maintain the activity of the RF-neurons and, in turn, hypoglossal motoneurons during wakefulness and NREM sleep. During REM sleep, raphe neurons become silent and, therefore, disinhibit the GABAergic neurons, which, in turn, increase inhibition of the RF-neurons. The increased inhibition of the RF-neurons and, at the same time, their disfacilitation due to silencing of A7 neurons result in the depression of both the RF-neuron activity and hypoglossal motoneurons, during REM sleep.

We also hypothesize that the mechanism of methysergide effects on REM-HD and the spontaneous activity in hypoglossal nerve in our antagonist experiments can be explained by the ability of methysergide to activate 5HT1A receptors (77). Consequently, methysergide diffused in the reticular formation and inhibited the local GABAergic neurons by activating their 5HT1A receptors; this disinhibited the RF-neurons and, in turn, hypoglossal motoneurons (see the time-course of methysergide effects in Figure 3). The disinhibitory effect of methysergide on REM-HD can be explained by the following mechanism: after methysergide binds and activates 5HT1A receptors on the local GABAergic neurons, the silencing of raphe neurons during REMSLS cannot disinhibit these GABAergic neurons because their inhibition is maintained by methysergide, thereby blocking the net inhibitory effect of 5HT on hypoglossal motoneurons during REM-HD.

The hypothesized presence of 5HT1A receptors on the local GABAergic neurons allows us to minimize the number of neuronal pools in the proposed circuit. In addition, this hypothesis is supported by the experimental observation that the systemically applied low doses of 8-OH-DPAT, a specific 5HT1A receptor agonist, readily silenced 5HT raphe neurons but do not affect the activity in hypoglossal nerve (78). Our explanation is that the 8-OH-DPAT inhibits the activity of 5HT raphe neurons via their own 5HT1A receptors. However, at the same time, the 8-OH-DPAT also occupies and activates the 5HT1A receptors that are located on the local GABAergic neurons (see Figure 4). The continuing activation of the GABAergic 5HT1A receptors keeps them inhibited despite the reduction of the 5HT drive from the silenced raphe neurons. Therefore, no change in the activity of hypoglossal nerve is observed.

To explain the excitatory effects of REM sleep on hypoglossal nerve activity, which was unmasked by bicuculline when it was present in the antagonist mix in our experiments, a hypothetical REM-ON excitatory drive has been added to the proposed circuit (see Figure 4). This excitatory drive activates hypoglossal motoneurons either directly or through the RF-neurons and it is controlled by GABAergic inhibition via GABA_A_ receptors that were blocked by bicuculline.

## Conclusion

The presented hypothetical neuronal circuit synthetizes and explains many experimental observations that were obtained up to date. The important new hypotheses that are introduced in this manuscript are the following: (1) the state-dependent control of hypoglossal motoneuron excitability is mediated by the RF-neurons that integrate the noradrenergic and serotonergic drives; (2) the noradrenergic disfacilitation during REM sleep is the key mechanism that is responsible for REM-HD; and (3) the serotonergic mechanisms involve local GABAergic neurons and contribute to REM-HD by a net inhibitory effect on the excitability of hypoglossal motoneurons. We believe that the outlined hypotheses and the proposed neuronal circuit will direct future experiments to advance our understanding of neurochemical mechanisms of REM-HD.

## Conflict of Interest Statement

The author declares that the research was conducted in the absence of any commercial or financial relationships that could be construed as a potential conflict of interest.
